# Common Genetic Variation in the Human *CTF1* Locus, Encoding Cardiotrophin-1, Determines Insulin Sensitivity

**DOI:** 10.1371/journal.pone.0100391

**Published:** 2014-07-15

**Authors:** Stefan Z. Lutz, Olga Franck, Anja Böhm, Jürgen Machann, Fritz Schick, Fausto Machicao, Andreas Fritsche, Hans-Ulrich Häring, Harald Staiger

**Affiliations:** 1 Department of Internal Medicine, Division of Endocrinology, Diabetology, Vascular Disease, Nephrology and Clinical Chemistry, University of Tübingen, Tübingen, Germany; 2 Institute for Diabetes Research and Metabolic Diseases of the Helmholtz Centre Munich at the University of Tübingen, Tübingen, Germany; 3 German Centre for Diabetes Research (DZD), Tübingen, Germany; 4 Department of Diagnostic and Interventional Radiology, Section on Experimental Radiology, Eberhard Karls University Tübingen, Tübingen, Germany; 5 Department of Internal Medicine, Division of Nutritional and Preventive Medicine, University of Tübingen, Tübingen, Germany; GDC, Germany

## Abstract

**Aims/Hypothesis:**

Recently, cardiotrophin-1, a member of the interleukin-6 family of cytokines was described to protect beta-cells from apoptosis, to improve glucose-stimulated insulin secretion and insulin resistance, and to prevent streptozotocin-induced diabetes in mice. Here, we studied whether common single nucleotide polymorphisms (SNPs) in the *CTF1* locus, encoding cardiotrophin-1, influence insulin secretion and insulin sensitivity in humans.

**Methods:**

We genotyped 1,771 German subjects for three *CTF1* tagging SNPs (rs1046276, rs1458201, and rs8046707). The subjects were metabolically characterized by an oral glucose tolerance test. Subgroups underwent magnetic resonance (MR) imaging/spectroscopy and hyperinsulinaemic-euglycaemic clamps.

**Results:**

After appropriate adjustment, the minor allele of *CTF1* SNP rs8046707 was significantly associated with decreased *in vivo* measures of insulin sensitivity. The other tested SNPs were not associated with OGTT-derived sensitivity parameters, nor did the three tested SNPs show any association with OGTT-derived parameters of insulin release. In the MR subgroup, SNP rs8046707 was nominally associated with lower visceral adipose tissue. Furthermore, the SNP rs1458201 showed a nominal association with increased VLDL levels.

**Conclusions:**

In conclusion, this study, even though preliminary and awaiting further confirmation by independent replication, provides first evidence that common genetic variation in *CTF1* could contribute to insulin sensitivity in humans. Our SNP data indicate an insulin-desensitizing effect of cardiotrophin-1 and underline that cardiotrophin-1 represents an interesting target to influence insulin sensitivity.

## Introduction

Diabetes, which is characterized by chronic hyperglycaemia, occurs either by developing insulin resistance and/or by beta-cell failure. Possible trigger for both are cytokines, which may be produced by the liver (hepatokines), skeletal muscle (myokines), or adipose tissue (adipokines). Beyond a large number of cytokines which have been shown to be involved as mediators in cellular insulin resistance and beta-cell failure often under inflammatory conditions, other cytokines are reported to exert beneficial effects with regard to improved glucose tolerance and insulin sensitivity [1]–[3].

Cardiotrophin-1 (CT-1) is a member of the interleukin-6 (IL-6) family of cytokines with a molecular mass of 21.5 kDa, which interacts with the glycoprotein 130 (gp130)/leukaemia inhibitory factor receptor (LIFR) heterodimer [4]. CT-1 was originally isolated from cardiac tissue and first described by its ability to protect cardiomyocytes from apoptosis but also to induce cardiac hypertrophy [5].

In several previous *in vitro* and mouse studies, CT-1 was shown to exert an important role in glucose and lipid metabolism. CT-1 knockout mice display mature-onset obesity, insulin resistance, and hypercholesterolemia. Moreover, recombinant CT-1 treatment corrected insulin resistance and reduced adiposity in ob/ob and high-fat diet (HFD) receiving mice, pointing to a key regulatory role of CT-1 in glucose and lipid metabolism [6]. Beside regenerative and anti-apoptotic functions in several tissues, e.g. motoneurons and hepatocytes [7], [8], in a recent study, CT-1 was reported to protect beta cells from apoptosis and to enhance glucose-stimulated insulin secretion, and additionally, to prevent streptozotocin-induced diabetes in a mouse model [9].

Some members of the IL-6 family like ciliary neurotrophic factor (CNTF) have been shown to improve insulin resistance and glucose tolerance by activating skeletal muscle AMPK [10]. Moreover, in a recent study, CT-1 was also referred to stimulate the oxidative metabolism through phosphorylation and activation of AMPK [6]. Thus, CT-1 is involved in AMPK signalling and represents a conceivable trigger for a cytokine-mediated improvement of insulin sensitivity and/or beta-cell survival and function.

These mouse and in vitro studies point to an important involvement of CT-1 in improving insulin secretion and insulin sensitivity. For this reason, and because of its high expression in tissues that play a pivotal role in the pathogenesis of type 2 diabetes like beta-cells, skeletal muscle and liver [6], [8], [9], the CT-1 gene (*CTF1)* would be a promising candidate to associate with diabetes, insulin secretion and insulin sensitivity. Thus, we asked whether genetic variation within or near *CTF1* has an impact on insulin secretion and insulin sensitivity in humans. To this end, we assessed in 1,771 German individuals at increased risk for type 2 diabetes, recruited from the Tübingen family (TÜF) study for type 2 diabetes, whether common single nucleotide polymorphisms (SNPs) with minor allele frequencies (MAFs) ≥0.05 tagging the human *CTF1* locus associate with prediabetic traits. Our data support a robust association of the *CTF1* SNP rs8046707 with insulin sensitivity in humans.

## Materials and Methods

### Ethics statement

The protocol of the study adhered to the Declaration of Helsinki and was approved by the local ethics board (Ethics Committee of the Medical Faculty of the Eberhard Karls University Tübingen). From all participants, informed written consent to the study was obtained.

### Subjects

An overall study group of 1,771 White European individuals from Southern Germany was recruited from the ongoing Tübingen Family study for type 2 diabetes (TÜF). This study group currently encompasses more than 2,700 participants at increased risk for type 2 diabetes (i.e., diagnosis of impaired fasting glycaemia, obesity, family history of type 2 diabetes, and/or previous gestational diabetes) [11]. All participants underwent the standard procedures of the protocol: assessment of medical history, routine blood analyses, alcohol and smoking status, physical examination, and oral glucose tolerance tests (OGTTs). The subjects were not on medication known to affect glucose tolerance, insulin sensitivity, or insulin secretion. 41 subjects were on lipid-lowering drugs (37 on statins, one on fibrates, two on ezetrol, and one on a combination of statins, fibrates, and ezetrol). From the overall study group, a subgroup of 312 subjects agreed to undergo magnetic resonance (MR) imaging and spectroscopy and another subgroup of 443 subjects a hyperinsulinaemic-euglycaemic clamp procedure. The clinical characteristics of the overall study group, the MR and the clamp subgroups are presented in Table 1.

**Table 1:** Clinical characteristics of the study groups.

	Overall study group	MR subgroup	ISI clamp subgroup
Sample size (N)	1,771	312	443
Women/men (%)	66.3/33.7	60.6/39.4	54.2/45.8
Age (y)	40±13	46±12	40±12
BMI (kg/m^2^)	29.9±8.9	29.9±5.1	27.4±5.6
Body fat (%)	28.6±9.7	32.7±8.7	28.6±9.7
Waist circumference (cm)	95.5±18.6	97.3±13.4	92.5±15.0
Fasting glucose (mmol/L)	5.15±0.53	5.26±0.50	5.02±0.54
Glucose 120 min OGTT (mmol/L)	6.36±1.61	6.93±1.57	6.22±1.70
Fasting insulin (pmol/L)	69.7±57.4	64.6±42.7	53.6±38.8
HOMA-IR (*10^-6^ mol*U*L^−2^)	2.73±2.41	2.55±1.82	2.05±1.65
ISI OGTT (*10^15^ L^2^*mol^−2^)	15.2±10.4	12.4±6.8	18.1±11.5
ISI Clamp (10*^6^ L*kg^−1^*min^−1^)	–	–	0.085±0.054
HOMA-B (U*mol^−1^)	144.5±119.3	126.8±80.8	124.9±102.1
AUC_Ins 0-30_/AUC_Glc0-30_ OGTT (*10^−9^)	45.0±32.7	42.9±27.7	37.3±24.2
AUC_C-Peptid 0-120_/AUC_Glc 0-120_ OGTT (*10^−9^)	318.7±103.3	307.1±90.9	310.7±97.7
Fasting FFA (µmol/L)	585.2±247.6	656.3±267.3	577.6±257.6
AUC_FFA_ (µmol/l)	480.9±214.4	526.6±186.6	452.0±197.7
Fasting triglycerides (mg/dL)^a^	119.0±78.2	124.4±98.5	114.4±88.6
Total cholesterol (mg/dL)^a^	192±37	194±36	191±36
LDL-cholesterol (mg/dL)^a^	118±33	122±30	118±31
HDL-cholesterol (mg/dL)^a^	54±14	52±13	56±15
Body fat (%)	32.8±12.1	32.7±8.7	28.6±9.7
Total adipose tissue (% BW)	–	30.2±9.0	–
Visceral adipose tissue (% BW)	–	3.32±1.71	–
Intrahepatic lipids (%)	–	5.97±6.51	–

Data are given as counts, percentages, or means ±SD. AUC - area under the curve; BMI - body mass index; BW - body weight; Glc - glucose; HOMA-IR - homeostasis model assessment of insulin resistance; Ins - insulin; ISI - insulin sensitivity index; MR - magnetic resonance; OGTT - oral glucose tolerance test; FFA - free fatty acid.

a Available in 1,628 participants of the overall study group.

### OGTT

A standard 75-g OGTT was performed after a 10 h-overnight fast. For the determination of plasma glucose, insulin, C-peptide, and free fatty acid (FFA) concentrations, venous blood samples were taken at time-points 0, 30, 60, 90, and 120 min [11].

### Hyperinsulinaemic-euglycaemic clamp

In subjects who agreed to undergo the hyperinsulinaemic-euglycaemic clamp, the clamp procedure was started after the 10-h overnight fast. The subjects received a primed infusion of insulin (40 mU*m^−2^*min^−1^) for 120 min, and glucose infusion was started to clamp the plasma glucose concentration at 5.5 mmol/L. For the measurement of plasma glucose, venous blood samples were drawn in 5-min intervals. Plasma insulin levels were determined at baseline and in the steady state of the clamp [11].

### Measurements of body fat content and body fat distribution

Waist circumference was measured in the upright position at the midpoint between the lateral iliac crest and the lowest rib (in cm). Body mass index (BMI) was determined as weight divided by height squared (kg/m^2^). In addition, the percentage of body fat was measured by bioelectrical impedance (BIA-101, RJL systems, Detroit, MI, USA). Total and visceral adipose tissue (TAT, VAT) contents were determined by whole-body MR imaging (% of body weight) [12]. The intrahepatic lipid content was determined by localized STEAM ^1^H-MR spectroscopy, as described earlier (% of signal) [13].

### Laboratory measurements

Plasma glucose was measured using a bedside glucose analyzer (glucose oxidase method, Yellow Springs Instruments, Yellow Springs, OH, USA) (mmol/L). Plasma insulin and C-peptide concentrations were measured by commercial chemiluminescence assays for ADVIA Centaur (Siemens Medical Solutions, Fernwald, Germany) (both pmol/L). Total-, high-density lipoprotein (HDL)-, and low-density lipoprotein (LDL)-cholesterol, and triglycerides were measured using the ADVIA 1800 clinical chemical analyzer. FFA concentrations were determined with an enzymatic method (WAKO Chemicals, Neuss, Germany).

### Selection of tagging SNPs

**Figure 1:**
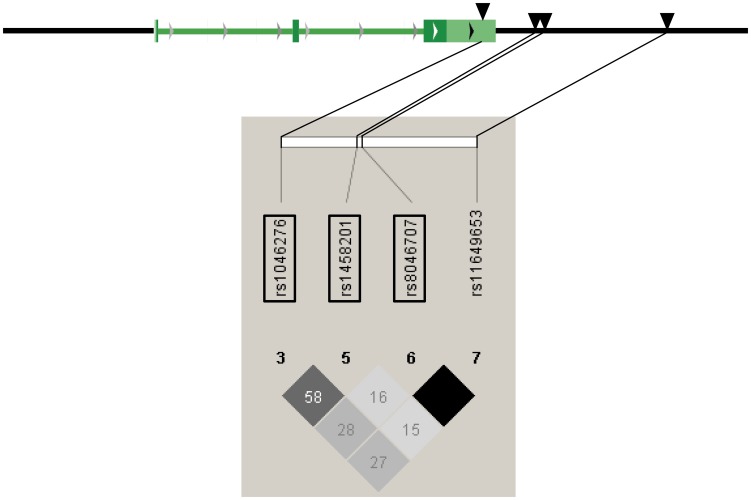
Genomic region of human chromosome 16p11.2 harbouring the *CTF1* gene and HapMap linkage disequilibrium (r^2^) data of common (MAF≥0.05) informative SNPs within this region. The *CTF1* gene consists of three exons and two introns and spans 6.953 kb from nucleotide 30,815,429 to nucleotide 30,822,381 (HapMap coordinates). The analyzed region additionally included 3 kb of the 5′-flanking region and 5 kb of the 3′-flanking region. The genotyped SNPs are highlighted by black frames. In the diamonds below the SNPs, r^2^ values are given (black diamonds: r^2^ = 1.0).

Based on publicly available phase III data of the International HapMap Project derived from Utah residents with Central European ancestry (release #28 August 2010, http://hapmap.ncbi.nlm.nih.gov/index.html.en), we screened in silico the complete *CTF1* gene spanning 6.953 kb (3 exons, 2 introns) on human chromosome 16p11.2 as well as 3 and 5 kb of its 5′- and 3′-flanking regions, respectively (Figure 1). Within this genomic locus, four informative SNPs with MAFs ≥0.05 (according to HapMap) were present: rs1046276 (C/T), rs1458201 (C/T), rs8046707 (G/A), and rs11649653 (C/G). The HapMap linkage disequilibrium (r^2^) data of these four common SNPs are schematically presented in Figure 1. Except SNP rs1046276, that is located in the 3′-untranslated region of exon 3, all SNPs reside in the 3′-flanking region of the gene. Since rs11649653 and rs8046707 were in complete linkage (r^2^ = 1.0) according to HapMap, only SNPs rs8046707, rs1046276, and rs1458201 were genotyped and further analyzed.

### Genotyping

In the TÜF study, DNA was isolated from the whole blood using a commercial DNA isolation kit (NucleoSpin, Macherey & Nagel, Düren, Germany). The three *CTF1* tagging SNPs were genotyped using the Sequenom mass ARRAY system with iPLEX software (Sequenom, Hamburg, Germany). The genotyping success rates were ≥99.8%. The Sequenom and TaqMan assays were validated by bidirectional sequencing in 50 randomly selected subjects, and both methods gave 100% identical results.

### Calculations

HOMA-IR was calculated as: c(glucose[mmol/L])_0_*c(insulin[mU/L])_0_, c = concentration [14]. The insulin sensitivity index derived from the OGTT (ISI OGTT) was calculated as formerly reported: 10,000/{c(glucose[mmol/L])_0_*c(insulin[pmol/L])_0_* c(glucose[mmol/L])_mean_*c(insulin[pmol/L])mean}^1/2^ [15]. The insulin sensitivity index derived from the hyperinsulinaemic-euglycaemic clamp (ISI clamp) was determined as glucose infusion rate to maintain euglycaemia during the last 20 min of the clamp (steady state) (µmol*kg^−1^*min^−1^) divided by the steady-state insulin concentration (pmol/L). HOMA-B was calculated as: {20* c(insulin[mU/L])_0_}/{c(glucose[mmol/L])_0_–3.5}. The OGTT-derived insulin release was calculated by AUC_Ins__0-30_/AUC_Glc__0-30_ and AUC_C-Pep__0-120_/AUC_Glc__0-120_, Ins = insulin (pmol/L), C-Pep = C-peptide (pmol/L), Glc = glucose (mmol/L). AUC_Ins__0-30_/AUC_Glc__0-30_ was calculated as: {c(insulin)_0_+c(insulin)_30_}/{c(glucose)_0_+c(glucose)_30_. AUC_C-Pep__0-120_/AUC_Glc__0-120_ was calculated by the trapezoid method: ½{½c(C-peptide)_0_+c(C- peptide)_30_+c(C-peptide)_60_+c(C-peptide)_90_+½c(C-peptide)_120_}/½{½c(glucose)_0_+c(glucose)_30_+ c(glucose)_60_+c(glucose)_90_+ ½c(glucose)_120_}. Both AUC_Ins__0-30_/AUC_Glc__0-30_ and AUC_C-Pep__0-120_/AUC_Glc__0-120_ were found to be superior to fasting state or OGTT-derived parameters for genetically determined beta-cell failure [16].

### Statistical analyses

Hardy-Weinberg equilibrium was tested by χ^2^ test. Linkage disequilibrium data (r^2^) between the SNPs were obtained with MIDAS 1.0 (http://www.genes.org.uk/software/midas). All continuous variables not normally distributed were log*_e_*-transformed prior to regression analysis. Multiple linear regression analysis was performed using the least-squares method with the trait of interest (measure of body fat content/distribution, glycaemia, insulin secretion, insulin sensitivity, or plasma lipid) as dependent variable, the SNP genotype (in the additive inheritance model) as independent variable, and gender, age, BMI, and insulin sensitivity as confounding variables where applicable. Based on the three non-linked SNPs tested, a p-value <0.0170 was considered statistically significant according to Bonferroni correction for multiple comparisons (α_corrected_ = 1-0.95^1/N^ with N = number of null hypotheses). We did not correct for the tested traits of interest since these were not considered as independent. The analyses were performed with the statistical software package JMP 8.0 (SAS Institute, Cary, NC, USA). In the dominant inheritance model, our overall study cohort was sufficiently powered (α<0.05; 1-β≥0.8) to detect effect sizes ≥0.13 (Cohen's d). Power calculations were performed using G*power 3.0 software available at http://www.psycho.uni-duesseldorf.de/aap/projects/gpower/.

## Results

### Clinical characteristics of the study groups

The overall population derived from the TÜF study consisted of 1,771 relatively young (median age 40 y) non-diabetic White European subjects with a median BMI of 29.9 kg/m^2^. Two thirds were women, one third men. The majority (∼71%) of the subjects were normal glucose tolerant (NGT), ∼29% were prediabetic: 11.2% had isolated impaired fasting glycaemia (IFG), 9.6% isolated impaired glucose tolerance (IGT), and 8.2% both IFG and IGT. The clinical characteristics of the overall population, MR and ISI clamp subgroups were largely comparable and given in Table 1. Though, the ISI clamp subgroup was somewhat more insulin-sensitive.

### Genotyping of *CTF1* tagging SNPs

The 1,771 study participants were genotyped for the SNPs rs1046276, rs1458201, and rs8046707 covering all common genetic variation in the *CTF1* gene locus with MAF ≥0.05 (Figure 1). All SNPs were in Hardy-Weinberg equilibrium (p≥0.1 all). The observed MAFs were 0.25 (rs1458201), 0.36 (rs1046276), and 0.41 (rs8046707) and were comparable to those provided by HapMap for the CEU population (0.21, 0.33, and 0.40, respectively). As expected from the HapMap data (Figure 1), the observed genetic linkage between the three SNPs was modest with r^2^ = 0.60 for the linkage between rs1046276 and rs1458201, r^2^ = 0.38 between rs1046276 and rs8046707, and r^2^ = 0.23 between rs1458201 and rs8046707.

### Genetic associations of *CTF1* with body fat content and body fat distribution

After adjustment for gender and age, none of the three tagging SNPs showed significant association (p>0.1) with parameters of body fat content (BMI, bioelectrical impedance-derived percentage of body fat, MR imaging-derived TAT) or body fat distribution (waist circumference, MR imaging-derived VAT, MR spectroscopy-derived intrahepatic lipids). After identical adjustment, the minor A-allele of SNP rs8046707 was nominally associated with reduced VAT (Table S1, p = 0.044).

### Genetic associations of *CTF1* with insulin sensitivity and glycaemia

**Figure 2:**
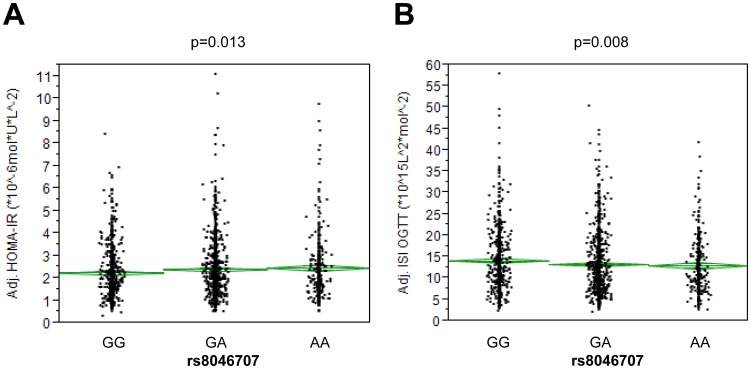
Association of SNP rs8046707 with HOMA-IR (A) and ISI OGTT (B). Data were adjusted for gender, age, and BMI. Diamonds represents means ± SE. HOMA-IR – homeostasis model assessment of insulin resistance; ISI OGTT – oral glucose tolerance test-derived insulin sensitivity index. SNP – single nucleotide polymorphism.

We then asked whether *CTF1* SNPs are associated with insulin sensitivity and/or glycaemia. After adjustment for gender, age and BMI, the minor A-allele of SNP rs8046707 was significantly associated with increased HOMA-IR (p = 0.013) and decreased ISI OGTT (p = 0.008) revealing an insulin-desensitizing effect of this allele (Figure 2 and Table 2). The SNP revealed effect sizes of 4.6% on HOMA-IR and -5.1% on ISI OGTT per allele. None of the other tested SNPs showed associations with insulin sensitivity and/or glycaemia (p>0.1).

**Table 2:** Associations between CTF1 SNPs and glycaemia and insulin sensitivity.

	Genotype	N overall	Fasting glc (mmol/L)	120 min glc OGTT (mmol/L)	AUC glc (mmol/L)	Fasting ins (pmol/L)	HOMA-IR (*10^−6^ mol*U*L^−2^)	ISI, OGTT (*10^15^ L^2^*mol^−2^)	N subgroup	ISI clamp (10^*6^ L*kg^−1^*min^−1^)
**rs1046276**	CC	732	5.16±0.54	6.34±1.64	14.88±3.21	70.73±57.91	2.79±2.45	14.82±10.19	182	0.087±0.057
	CT	794	5.14±0.52	6.39±1.60	14.76±3.05	69.33±57.33	2.71±2.37	15.24±10.52	185	0.083±0.052
	TT	245	5.11±0.56	6.35±1.57	14.70±3.01	68.05±56.29	2.67±2.40	15.86±10.87	76	0.085±0.051
**p_add_**	–	–	0.325	0.502	0.555	0.325	0.104	0.11	–	0.392
**rs1458201**	CC	988	5.16±0.54	6.36±1.65	14.85±3.19	69.75±56.69	2.75±2.40	15.01±10.25	240	0.084±0.054
	CT	666	5.12±0.51	6.34±1.56	14.67±3.01	69.76±58.39	2.71±2.37	15.36±10.74	171	0.085±0.052
	TT	117	5.18±0.58	6.55±1.58	15.11±3.01	69.39±58.26	2.79±2.65	15.25±10.19	32	0.088±0.060
**p_add_**	–	–	0.459	0.371	0.989	0.459	0.453	0.464	–	0.875
**rs8046707**	GG	624	5.13±0.56	6.34±1.60	14.67±3.09	68.43±56.85	2.68±2.40	15.72±10.75	171	0.083±0.049
	GA	858	5.16±0.50	6.43±1.62	14.87±3.02	70.93±57.76	2.78±2.40	14.76±10.39	189	0.086±0.058
	AA	289	5.15±0.59	6.21±1.61	14.86±3.44	68.98±57.64	2.71±2.45	15.10±9.80	83	0.085±0.053
**p_add_**	–	–	0.347	0.945	0.122	0.347	0.013*	0.008*	–	0.414

Data represents means ± SD. Prior to statistical analysis, glucose concentrations and insulin senstitivity measures were adjusted for gender, age and BMI. Nominal associations marked by bold fonts. AUC - area under the curve; glc - glucose; HOMA-IR - homeostasis model assessment of insulin resistance; ins - insulin; ISI - insulin sensitivity index; OGTT - oral glucose tolerance test.

### Genetic associations of *CTF1* with insulin release

After adjustment of gender, age, BMI and ISI OGTT, none of the tagging SNPs was significantly or nominally associated with OGTT-derived parameters of insulin release (p>0.3) as given in Table S2.

### Genetic associations of *CTF1* with indices of lipid metabolism

From 1,628 subjects of the overall group, quantitative measurements of triglycerides (TG), total-, LDL-, and HDL-cholesterol were available. The number of the subjects receiving lipid-lowering drugs is denoted in the Materials and Methods section. An additional adjustment for the drug classes by introducing appropriate dummy variables in the regression models was carried out. In our study, the minor T-allele of SNP rs1458201 was nominally associated with increased VLDL levels (p = 0.024) (Table S3).

### Interrogation of MAGIC data for replication

We screened the MAGIC data from 37,037 non-diabetic subjects of European descent (HOMA-IR dataset) to replicate the effects of SNP rs8046707 on HOMA-IR [17]. However, SNP rs8046707 was not depicted on the arrays used by MAGIC. Therefore, we looked for proxies for SNP rs8046707 to investigate whether they show associations with fasting insulin and HOMA-IR. Using SNAP (http://www.broadinstitute.org/mpg/snap/ldsearch.php), we found two proxies, rs11649653 (r^2^ = 0.967) and rs4889603 (r^2^ = 0.870). They showed no significant association with fasting insulin (p = 0.262 and p = 0.285, respectively) or HOMA-IR (p = 0.171 and p = 0.196, respectively). Two other proxies, rs12917722 and rs11640961, were not depicted on the arrays.

## Discussion

Up to now, several different studies tried to highlight the role of CT-1 with regard to metabolic disorders *in vitro* as well as *in vivo*, both in murine models and in humans. Among a variety of metabolic and cytoprotective activities reported so far, CT-1 has been shown to play an important protective role in beta-cell viability and to improve glucose-stimulated insulin secretion, and moreover, to prevent streptozotocin-induced diabetes in mice [9]. In addition, recombinant CT-1 treatment corrected insulin resistance and reduced adiposity in ob/ob and high-fat diet receiving mice, pointing to a key regulatory role of CT-1 in insulin sensitivity and lipid metabolism [6].

In this study, we report a significant insulin-desensitizing effect of the minor A-allele of *CTF1* SNP rs8046707. After adjustment for gender, age and BMI, the minor A-allele of SNP rs8046707 was significantly associated with increased HOMA-IR and reduced ISI OGTT. However, we could not confirm these results in the ISI clamp subgroup, as there was no association either of the SNP rs8046707 or of the other two analyzed common variants with hyperinsulinaemic-euglycaemic clamp-derived insulin sensitivity parameters. One possible reason for this discrepancy may be due to the limited sample size of this subgroup. Of interest, the ISI clamp subgroup showed somewhat higher insulin sensitivity compared to the overall group, so that a possible selection bias can not be excluded. We hypothesize that the more insulin sensitive subgroup possess protective compensatory mechanisms to counteract the adverse effects of the minor A-allele of *CTF1* SNP rs8046707, while the more insulin resistant overall group may lack these compensatory mechanisms. Otherwise, ISI clamp-derived parameters mainly reflect skeletal muscle specific insulin sensitivity, whereas elevated HOMA-IR is commonly due to hepatic insulin resistance. Hence, the isolated detection of reduced insulin sensitivity by HOMA-IR and ISI OGTT, without corresponding alterations in ISI clamp-derived indices, could reflect a selective hepatic insulin resistance in minor A-allele carriers of SNP rs8046707. Thus, both in the overall study group and the ISI clamp subgroup neither fasting glucose nor fasting insulin were associated with *CTF1* variations. It would be interesting to carry out further replications of our results in larger comparably phenotyped study populations.

Our finding that the *CTF1* SNP rs8046707 determines insulin sensitivity, appears in good agreement with previous *in vitro* and *in vivo* mouse studies, reporting on CT-1 mediated enhanced insulin signaling in muscle and adipocyte and an amelioration of insulin resistance in obese mice after chronic administration of CT-1 [6]. Asrih et al. reported a concentration-dependent dual effect of CT-1 on insulin-stimulated glucose transport in cardiomyocytes, with stimulated glucose transport at high concentrations CT-1 (10 nM) and inhibited glucose transport at low concentrations (1 nM), also pointing to an involvement of CT-1 in insulin sensitivity [18]. The first finding at low CT-1 concentrations was proposed to be due to a reduction of GLUT-4 expression and, concomitantly, reduced insulin signaling due to enhanced SOCS-3 expression, whereas this inhibitory mechanism seemed to be overridden at high CT-1 concentrations.

Several members of the IL-6 family like ciliary neurotrophic factor (CNTF) have been shown to improve insulin resistance and glucose tolerance by activating skeletal muscle AMPK [10] and further, in a recent study, CT-1 itself was also found to stimulate the oxidative metabolism through phosphorylation and activation of AMPK [6]. Interestingly, an insulin-independent effect of CT-1 on glucose uptake is likely, based on the study mentioned before, showing an AMPK activation through calmodulin-dependent kinase II after exposure to high concentrations of CT-1 [18], but also on the study reported by Chopra et al. demonstrating AMPK to phosphorylate the insulin receptor independently of insulin [19]. Hence, CT-1 is involved in AMPK signalling and, in agreement with our data, it may represent a conceivable trigger for a cytokine-mediated improvement of insulin sensitivity.

On the other hand, several previous *in vitro* and *in vivo* reports demonstrate also an involvement of CT-1 in insulin sensitivity, though, a deleterious effect of CT-1 on the development of insulin resistance [20]. Moreover, some studies point to a positive correlation between upregulation of CT-1 and impaired fasting glucose (IFG), hyperglycemia, newly diagnosed DM or obesity [21]–[23]. As already mentioned, CT-1 may exert opposite effects in insulin stimulated glucose uptake depending on the CT-1 concentration [18]. As previously discussed [24], based on mouse studies, elevated CT-1 plasma levels in obesity may represent a potential protective way to antagonize the deleterious metabolic dysregulations. For the moment, it is unclear whether CT-1 exerts similar biochemical features in humans and in mice, and this could be a possible explanation for the different results regarding insulin sensitivity between the mentioned studies above.

Surprisingly, our findings with regard to insulin release do not support the previous mouse study results of Jiménez-Gonzales, who reported CT-1 to protect beta cells from apoptosis and to enhance glucose-stimulated insulin release [9]. In our study, we didńt find an impact of the three investigated *CTF1* SNPs on insulin secretion. One possible explanation might be a smaller impact of *CTF1* polymorphisms in humans on CT-1 plasma levels compared to the used CT-1 concentrations in the *in vitro* experiments. In addition, perhaps more relevantly, a germline knockout mouse with potentially massive genetic variation may display a diverse phenotype than humans with expectedly weaker alterations due to common variations in the *CTF1* gene.

Beside significant associations with insulin sensitivity markers, we also found a nominal association between reduced VAT and SNP rs8046707 (p = 0.044). Up to now, several groups reported an impact of CT-1 on obesity. In a previous study, recombinant CT-1 reversed obesity, due to reduction of fat stores and remodeling of white adipose tissue, insulin resistance and DM in *ob/ob* and high-fat diet fed mice, related to an increase in energy expenditure and decreased food intake [6]. However, because of the weak nominal association, without achieving significance after Bonferroni correction, and moreover, because of the controversy, that the minor A-allele of SNP rs8046707, which associates with significantly reduced insulin sensitivity in our study, was concomitantly nominally associated with decreased VAT, we think that this result may represent a chance finding due to the limited number of subjects included in the MR subgroup needing further replication in larger study populations.

Although CT-1 was reported to be upregulated in human and murine steatotic livers and, chronic CT-1 treatment to reverse hepatic steatosis in obese mice after AMPK activation [25], we could not detect any involvement of the three SNPs in intrahepatic lipid content. Finally, in our study, SNP rs1458201 showed a nominal association with increased VLDL levels. With regard to the small study population used for this measurement, this result should be replicated in a similarly well characterized study population.

A limitation of the study could be the comparatively small number of subjects included in the ISI clamp subgroup, for which reason we probably could not confirm the significant HOMA-IR- and ISI OGTT-associations with SNP rs8046707, as found in the overall study group. Furthermore, only relatively young (median age 40 y) White European subjects were included in this study, so that the impact of *CTF1* SNPs on insulin sensitivity and other related metabolic traits in elderly or other ethnical groups is not assessable based on this study. In addition, due to the limited number of participants of the TÜF study, only common variants with MAFs ≥0.05 were analyzed, so that additional rare SNPs with direct effect on CT-1 may exist. Finally, we could not replicate our results in the MAGIC dataset (using two closely linked SNPs as proxies), which may have several reasons. First, the HOMA-IR data from the 37,037 non-diabetic subjects were not adjusted for BMI, as compared to our data. Second, the MAGIC dataset is a result of meta-analyses of 21 genome-wide association studies, with a considerable heterogeneity, e.g., with respect to age of the participants.

In conclusion, the present study indicates that the *CTF1* gene, encoding the cytokine cardiotrophin-1, could be involved in the control of insulin sensitivity in humans. This cytokine therefore represents a promising target to influence insulin resistant states in diabetes.

## Supporting Information

Table S1 Associations between CTF1 SNPs and parameters of body fat content/distribution. Data represents means±SD. Prior to statistical analysis, all measures were adjusted for gender and age. Nominal associations marked by bold fonts. BMI - body mass index; Waist - waist circumference; TAT - total adipose tissue; VAT - visceral adipose tissue; IHL - intrahepatic lipids.
(DOC)

Table S2 Associations between CTF1 SNPs and insulin secretion. Data represents means±SD. Prior to statistical analysis, indices of insulin secretion were adjusted for gender, age, BMI and ISI OGTT. AUC - area under the curve; Glc - glucose; Ins - insulin; HOMA-B - homeostasis model assessment of beta-cell function; ISI - insulin sensitivity index; OGTT - oral glucose tolerance test.
(DOC)

Table S3 Associations between CTF1 SNPs and indices of lipid metabolism. Data represents means±SD. Prior to statistical analysis, indices of lipid metabolism were adjusted for gender, age, BMI and lipid-lowering medication. AUC - area under the curve; TG, triglyceride; Chol, cholesterol; LDL, low-density lipoprotein; HDL, high-density lipoprotein; VLDL, very low-density lipoprotein.
(DOC)
