# Supplementary material for: Common Genetic Variation in the Human CTF1 Locus, Encoding Cardiotrophin-1, Determines Insulin Sensitivity
Source: PLoS One. 2014 Jul 15;9(7):e100391. doi: 10.1371/journal.pone.0100391 (PMC4099130; doi:10.1371/journal.pone.0100391)
Supplement: Table S1 — Associations between CTF1 SNPs and parameters of body fat content/distribution. Data represents means±SD. Prior to statistical analysis, all measures were adjusted for gender and age. Nominal associations marked by bold fonts. BMI - body mass index; Waist - waist circumference; TAT - total adipose tissue; VAT - visceral adipose tissue; IHL - intrahepatic lipids. (DOC) [file pone.0100391.s001.doc]

**Table S1. Associations between CTF1 SNPs and parameters of body fat content/distribution.**

|  | **Genotype** | **N overall** | **BMI** | **Body fat (%)** | **Waist (cm)** | **N subgroup** | **TAT (%BW)** | **VAT (%BW)** | **IHL (%)** |
| --- | --- | --- | --- | --- | --- | --- | --- | --- | --- |
| **rs1046276** | CC | 732 | 29.9±8.9 | 32.3±11.8 | 96±19 | 132 | 30.1±9.4 | 3.42±1.74 | 6.40±6.71 |
|  | CT | 794 | 29.9±8.8 | 33.2±12.4 | 95±19 | 127 | 30.7±9.1 | 3.21±1.60 | 5.61±6.39 |
|  | TT | 245 | 30.0±8.9 | 32.9±12.1 | 96±18 | 53 | 29.6±8.1 | 3.34±1.91 | 5.79±6.36 |
| p_add_ | – | – | 0.927 | 0.828 | 0.769 | – | 0.903 | 0.797 | 0.377 |
| **rs1458201** | CC | 988 | 30.0±9.0 | 32.5±12.0 | 96±19 | 174 | 30.3±9.2 | 3.38±1.70 | 6.20±6.72 |
|  | CT | 666 | 29.8±8.6 | 33.0±12.4 | 96±18 | 119 | 30.1±8.9 | 3.30±1.74 | 5.81±6.34 |
|  | TT | 117 | 30.4±9.3 | 33.8±11.8 | 96±17 | 19 | 30.9±8.2 | 2.97±1.71 | 4.92±5.68 |
| p_add_ | – | – | 0.757 | 0.516 | 0.627 | – | 0.943 | 0.491 | 0.206 |
| **rs8046707** | GG | 624 | 30.2±9.1 | 33.2±12.5 | 96±19 | 123 | 30.4±8.3 | 3.34±1.80 | 5.91±6.44 |
|  | GA | 858 | 30.0±8.9 | 33.2±12.1 | 96±19 | 139 | 30.8±9.4 | 3.27±1.63 | 6.07±6.84 |
|  | AA | 289 | 29.0±8.1 | 30.7±11.1 | 95±18 | 50 | 28.3±9.5 | 3.43±1.74 | 5.87±5.84 |
| p_add_ | – | – | 0.12 | 0.429 | 0.203 | – | 0.486 | **0.044** | 0.732 |

Data represents means±SD. Prior to statistical analysis, all measures were adjusted for gender and age. Nominal associations marked by bold fonts. BMI - body mass index; Waist - waist circumference; TAT - total adipose tissue; VAT - visceral adipose tissue; IHL - intrahepatic lipids.
