# Supplementary material for: Common Genetic Variation in the Human CTF1 Locus, Encoding Cardiotrophin-1, Determines Insulin Sensitivity
Source: PLoS One. 2014 Jul 15;9(7):e100391. doi: 10.1371/journal.pone.0100391 (PMC4099130; doi:10.1371/journal.pone.0100391)
Supplement: Table S2 — Associations between CTF1 SNPs and insulin secretion. Data represents means±SD. Prior to statistical analysis, indices of insulin secretion were adjusted for gender, age, BMI and ISI OGTT. AUC - area under the curve; Glc - glucose; Ins - insulin; HOMA-B - homeostasis model assessment of beta-cell function; ISI - insulin sensitivity index; OGTT - oral glucose tolerance test. (DOC) [file pone.0100391.s002.doc]

**Table S2. Associations between CTF1 SNPs and insulin secretion.**

|  | **Genotype** | **N overall** | **HOMA-B (U*mol^-1^)** | **AUC_Ins 0-30_/AUC_Glc 0-30_ (*10^-9^)** | **AUC_C-Peptid 0-120_/AUC_Glc 0-120_ (*10^-9^)** |
| --- | --- | --- | --- | --- | --- |
| **rs1046276** | CC | 732 | 146.29±119.04 | 44.89±30.76 | 320.24±101.23 |
|  | CT | 794 | 143.61±118.73 | 45.29±33.64 | 316.42±103.39 |
|  | TT | 245 | 142.07±122.37 | 44.67±35.13 | 321.80±109.46 |
| p_add_ | – | – | 0.943 | 0.898 | 0.878 |
| **rs1458201** | CC | 988 | 144.08±114.96 | 44.68±30.51 | 317.40±100.04 |
|  | CT | 666 | 146.21±128.62 | 46.11±35.83 | 321.72±106.50 |
|  | TT | 117 | 138.45±99.37 | 41.95±31.68 | 313.16±112.48 |
| p_add_ | – | – | 0.868 | 0.327 | 0.963 |
| **rs8046707** | GG | 624 | 143.20±121.19 | 44.81±34.93 | 317.26±106.16 |
|  | GA | 858 | 144.99±117.09 | 45.37±31.23 | 319.85±101.27 |
|  | AA | 289 | 145.89±122.12 | 44.55±31.99 | 318.69±103.50 |
| p_add_ | – | – | 0.683 | 0.938 | 0.967 |

Data represents means±SD. Prior to statistical analysis, indices of insulin secretion were adjusted for gender, age, BMI and ISI OGTT. AUC - area under the curve; Glc - glucose; Ins - insulin; HOMA-B - homeostasis model assessment of beta-cell function; ISI - insulin sensitivity index; OGTT - oral glucose tolerance test.
