# Supplementary material for: Common Genetic Variation in the Human CTF1 Locus, Encoding Cardiotrophin-1, Determines Insulin Sensitivity
Source: PLoS One. 2014 Jul 15;9(7):e100391. doi: 10.1371/journal.pone.0100391 (PMC4099130; doi:10.1371/journal.pone.0100391)
Supplement: Table S3 — Associations between CTF1 SNPs and indices of lipid metabolism. Data represents means±SD. Prior to statistical analysis, indices of lipid metabolism were adjusted for gender, age, BMI and lipid-lowering medication. AUC - area under the curve; TG, triglyceride; Chol, cholesterol; LDL, low-density lipoprotein; HDL, high-density lipoprotein; VLDL, very low-density lipoprotein. (DOC) [file pone.0100391.s003.doc]

**Table S3. Associations between CTF1 SNPs and indices of lipid metabolism.**

|  | **Genotype** | **N overall** | **TG (mg/dL)** | **Fasting FFA (mmol/L)** | **AUC FFA (mmol/L)** | **Chol (mg/dL)** | **LDL (mg/dL)** | **HDL (mg/dL)** | **N subgroup** | **VLDL (mg/dL)** |
| --- | --- | --- | --- | --- | --- | --- | --- | --- | --- | --- |
| **rs1046276** | CC | 667 | 123.67±88.87 | 586.78±234.24 | 484.72±203.03 | 192.14±37.01 | 118.05±32.81 | 53.93±14.28 | 53 | 9.36±8.33 |
|  | CT | 735 | 116.13±68.00 | 582.03±266.56 | 470.31±198.84 | 192.05±36.35 | 118.63±32.35 | 54.49±14.74 | 58 | 12.86±12.18 |
|  | TT | 226 | 114.67±75.40 | 579.48±218.07 | 474.06±204.21 | 191.45±38.25 | 118.47±32.50 | 54.45±13.43 | 24 | 10.67±7.08 |
| p_add_ | – | – | 0.2 | 0.27 | 0.08 | 0.96 | 0.47 | 0.52 | – | 0.09 |
| **rs1458201** | CC | 903 | 121.81±86.41 | 585.90±254.57 | 480.64±201.02 | 191.56±36.51 | 117.87±32.18 | 53.92±14.32 | 71 | 9.46±8.64 |
|  | CT | 616 | 114.77±66.43 | 578.78±240.39 | 468.72±199.16 | 192.13±36.93 | 118.67±32.60 | 54.71±14.69 | 53 | 13.40±12.02 |
|  | TT | 109 | 119.91±67.34 | 592.13±223.32 | 489.71±215.47 | 195.00±39.62 | 120.82±35.27 | 54.45±12.99 | 11 | 10.55±5.92 |
| p_add_ | – | – | 0.88 | 0.66 | 0.31 | 0.56 | 0.49 | 0.37 | – | **0.024** |
| **rs8046707** | GG | 572 | 115.14±69.79 | 588.76±278.12 | 472.94±203.10 | 191.71±35.35 | 118.40±31.54 | 54.30±13.72 | 48 | 11.58±11.22 |
|  | GA | 797 | 118.25±69.77 | 583.69±225.35 | 483.73±203.80 | 191.16±36.61 | 118.33±32.28 | 54.12±14.76 | 63 | 10.74±9.13 |
|  | AA | 259 | 129.93±112.45 | 572.07±239.25 | 463.59±189.08 | 195.27±40.71 | 118.43±35.50 | 54.57±14.58 | 24 | 11.04±10.32 |
| p_add_ | – | – | 0.15 | 0.42 | 0.18 | 0.48 | 0.52 | 0.86 | – | 0.6 |

Data represents means±SD. Prior to statistical analysis, indices of lipid metabolism were adjusted for gender, age, BMI and lipid-lowering medication. AUC - area under the curve; TG, triglyceride; Chol, cholesterol; LDL, low-density lipoprotein; HDL, high-density lipoprotein; VLDL, very low-density lipoprotein.
